# Application of Ni(II)-Assisted Peptide Bond Hydrolysis to Non-Enzymatic Affinity Tag Removal

**DOI:** 10.1371/journal.pone.0036350

**Published:** 2012-05-04

**Authors:** Edyta Kopera, Agnieszka Belczyk-Ciesielska, Wojciech Bal

**Affiliations:** Institute of Biochemistry and Biophysics, Polish Academy of Sciences, Warsaw, Poland; University of Cambridge, United Kingdom

## Abstract

In this study, we demonstrate a non-enzymatic method for hydrolytic peptide bond cleavage, applied to the removal of an affinity tag from a recombinant fusion protein, SPI2-SRHWAP-His_6_. This method is based on a highly specific Ni(II) reaction with (S/T)XHZ peptide sequences. It can be applied for the protein attached to an affinity column or to the unbound protein in solution. We studied the effect of pH, temperature and Ni(II) concentration on the efficacy of cleavage and developed an analytical protocol, which provides active protein with a 90% yield and ∼100% purity. The method works well in the presence of non-ionic detergents, DTT and GuHCl, therefore providing a viable alternative for currently used techniques.

## Introduction

Current techniques of protein science often require significant amounts of pure recombinant proteins. The affinity technology delivers high quantities of recombinant proteins for such studies. However, many applications require the removal of affinity tags after the purification step, since the tags often change conformation of proteins, alter their biological activity, or make them toxic [1]. Protease-mediated cleavage used commonly for this purpose has two serious drawbacks: non-specific digestion of the target protein is common and the protease must be removed after cleavage, thus requiring additional purification steps. Moreover, proteolytic enzymes are often expensive and thus not feasible for large-scale use. The development of self-cleaving affinity tags helped overcome some of these limitations. These tags consist of an autoprocessing domain fused to the affinity tag, enabling one-step purification. Those most commonly utilized for recombinant protein purification include inteins [2], the catalytic core of sortase A [3], and FrpC protein [4]. In this case, the cleavage process is strictly dependent on the preservation of the native self-cleaving domain conformation, which narrows requirements for reaction conditions. Also, large sizes of these moieties constitute disadvantage, diminishing the solubility and purification efficiency.

Chemical cleavage agents have been considered an inexpensive alternative for biological methods, but none has been found to exhibit sufficient sequence specificity and all produce high levels of by-products, due to harsh reaction conditions. This also comprises metal ions and metal complexes [5]–[8]. One promising attempt was the specific hydrolysis of the –DKTH- and –DKSH- peptide sequences by Cu(II) ions to cleave an immunoglobulin [9], [10]. This method has two distinct disadvantages. The Cu(II)-related hydrolysis occurred before the T/S residues. Thus, two additional amino acids (DK) were incorporated to the target protein as a consequence of the reaction specificity. Moreover, multiple unspecific reactions should be expected for this method, due to the ability of Cu(II) complexes to generate reactive oxygen species. Also the [Pd(en)(H_2_O)_2_]^2+^ complex was used to remove the affinity tag from an engineered protein. A Cys-His unit was introduced between cecropin CMIV and its N-terminal GST fusion partner for this purpose. After incubation the cleavage at the His-Arg bond was observed. These preliminary results held some promise of an application of this Pd(II) complex as cleavage agent for the production of recombinant proteins, but its sequence specificity did not seem to be sufficiently high for universal applications [11].

Our previous studies demonstrated that Ni(II) ions can hydrolyze the peptide bond preceding the serine or threonine in S/T-X-H-Z sequences. X and Z can be any amino acid residues, except of X = proline, but efficient reaction could only be obtained for several bulky/hydrophobic substitutions in these positions. Such tetrapeptide specificity of the cleavage site is similar to those of proteolytic enzymes. Using a library of model peptides and subsequent detailed kinetic studies we characterized the kinetics of this hydrolytic reaction and its molecular mechanism [12]–[17]. The crucial steps of the reaction include: the formation of the square-planar active complex with the Ni(II) ion bonded by the imidazole nitrogen and three preceding amide nitrogens (4N complex), the N-O acyl shift involving the hydroxyl group of the Ser or Thr residue, the formation of an ester intermediate and finally the spontaneous hydrolysis of this ester in the presence of water. Interestingly, a similar mechanism of sequence-specific peptide bond cleavage is used by Nature (e.g. Hedgehog protein, inteins) as the first step of the protein splicing process [18], [19].

In order to verify the biotechnological applicability of this Ni(II)-assisted reaction we use the recombinant SPI2 protein extended C-terminally by the SRHWAP-His_6_ dodecapeptide, which comprises the best Ni(II)-sensitive tetrapeptide (SRHW), obtained by peptide library screening [16], linked to the His-tag domain. SPI2 is a structurally unique Kazal-type proteinase inhibitor identified in the silk of wax moth *Galleria mellonella* (Lepidoptera). It is the shortest Kazal-type inhibitor in animals. SPI2 exhibited high activity against bacterial and fungal proteinases [20]. This robust, small, well characterized recombinant protein, is an appropriate test object for the method development, as its biological activity after the purification procedure can be easily confirmed by a protease inhibition test [21].

## Results

The SRHWAP peptide was cloned between the SPI2 protein and the C-terminal hexahistidine affinity tag. The SPI2-SRHWAP-His_6_ fusion protein secreted to the medium was purified by affinity chromatography on Ni-NTA agarose. After elution and dialysis, the fusion protein was further purified by HPLC and lyophilised. This purification procedure was applied in order to obtain the precise amounts of the fusion protein for quantitative studies of the Ni(II)-dependent tag removal reaction. The experiments were carried out in a buffer solution, as well as on a Ni-NTA agarose column.

### In-solution cleavage

**Figure 1:**
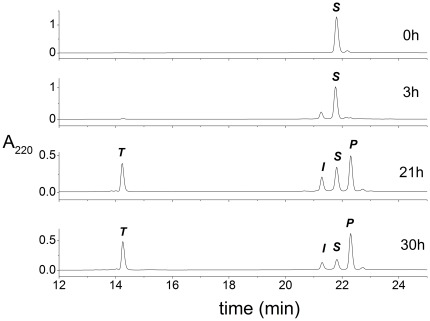
The examples of chromatograms of reaction mixture containing initially 20 µM SPI2-SRHWAP-His_6_ fusion protein, 0.5 mM NiCl_2_ and 100 mM Hepes buffer, pH 8.2 incubated and 45°C. Incubation times are indicated on the plot. Peak labels denote reaction substrate and products, identified using ESI-MS: ***S***, substrate (5868 Da); ***P***, pure SPI2 protein (4310 Da); ***T***, the SRHWAP-His_6_ tag (1574 Da); ***I***, the intermediate product of hydrolysis (5868 Da).

**Figure 2:**
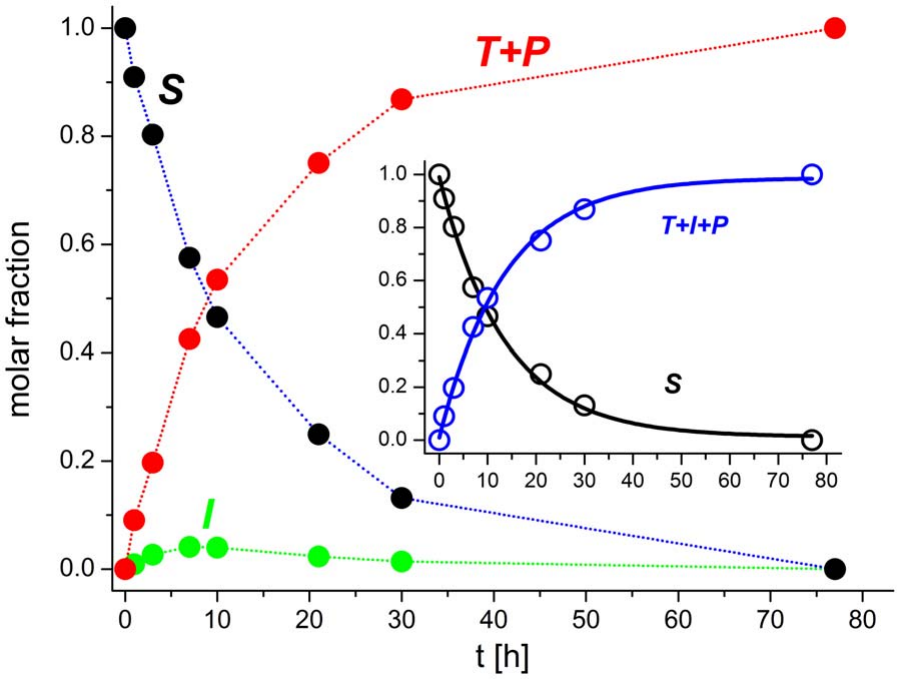
The example of hydrolysis rate constant calculation using the data illustrated in **Fig. 1**. The main plot shows peak integrals normalized to molar fractions of the initial SPI2-SRHWAP-His_6_ fusion protein concentration, and the inset presents 1st order rate constant fits to the substrate decay and product formation. Species are labeled according to Fig. 1.

In a preliminary experiment, samples of 20 µM SPI2-SRHWAP-His_6_ fusion protein were incubated with 0.5 mM Ni(II) ions in 100 mM Hepes buffer at pH 8.2 and 45°C. For the kinetic measurements the aliquots were periodically removed from the thermoblock and the hydrolysis products were analyzed by HPLC. The controls without Ni(II) were incubated and analyzed in the same way. Fig. 1 presents examples of chromatograms. All peaks were collected and their molecular masses measured using ESI-MS. Masses analysed agreed with theoretical ones and assigned to the substrate (in brief ***S***, 5868 Da), the pure SPI2 protein (***P***, 4310 Da) and the cleaved-off tag SRHWAP-His_6_ (***T***, 1574 Da). The molecular mass of the fourth peak was exactly the same as for the substrate. However, the differences in retention times of these two peaks indicate different hydrophobicities of the respective molecules. Further analysis (see below) identified this peak as the intermediate reaction product (in brief, ***I***) Fig. 2 presents the changes in the ***S, I, P*** and ***T*** amounts observed during the incubation.

No unspecific cleavage of the target protein was observed within the detection limits of the HPLC UV detector, roughly estimated as lower than 0.01% of the initial protein, even after prolonged incubation.

In all experiments we noted the presence of low amounts of ***I***. We calculated the kinetic constants of Ni(II)-dependent peptide hydrolysis by observing both the growth of the peaks corresponding to the protein products and the decrease of the peaks of the substrate. Despite the presence of ***I***, both obeyed the 1^st^ order rate law sufficiently well (Fig. 2). The hydrolysis rates were obtained by the fitting of peak integrals to the exponential (1^st^ order) rate equation. The HPLC injection errors were eliminated by normalization of peak integrals separately for each injection. Such normalized data were used in calculations, as illustrated in the inset to Fig. 2.

**Figure 3:**
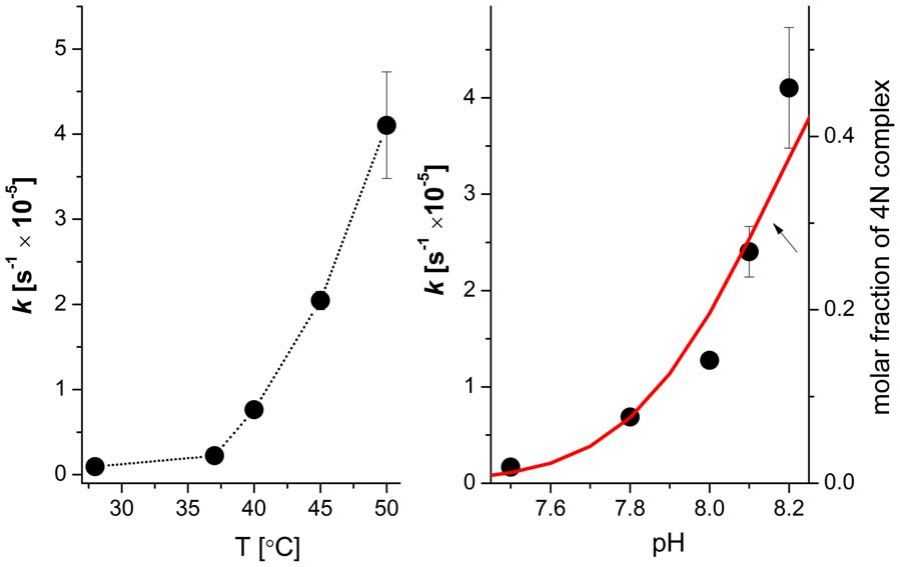
The hydrolysis rate constants of 20 µM SPI2-SRHWAP-His_6_ fusion protein in the presence of 0.5 mM NiCl_2_ and 100 mM Hepes buffer. A, temperature dependence; B, pH dependence. The red line indicates the molar fraction of the hydrolytic Ni(II) complex of the Ac-GASRHWKFL-NH_2_ peptide [17], calculated for 20 µM peptide and 0.5 mM Ni(II).

The tag removal reaction was studied at 50, 45, 40, 37, and 28°C (Table 1, Fig. 3) and, for 50°C, at pH 8.2, 8.1, 8.0, 7.8, and 7.5 (Table 2, Fig. 3). In all cases, the process of hydrolysis could be reliably analyzed using a 1^st^ order rate constant. As in peptide studies [16], [17] the reaction rate accelerated significantly with the increase of pH and the temperature.

**Table 1:** The temperature dependence of the 1^st^ order rate constant and reaction half-times for SPI2-SRHWAP-His_6_ hydrolysis in solution, at H 8.2, determined by HPLC.

T [°C]	k ± S.D. (s^−1^×10^−5^)	t_1/2_ (h)
**50**	4.10±0.63	5
**45**	2.05±0.11	9
**40**	0.76±0.08	25
**37**	0.22±0.07	86
**28**	0.09±0.005	206

**Table 2:** The pH dependence of the 1^st^ order rate constant and reaction half-time for SPI2-SRHWAP-His_6_ hydrolysis in solution, at 50°C, determined by HPLC.

pH	k ± S.D. (s^−1^×10^−5^)	t_1/2_ (h)
**7.5**	0.166±0.03	116
**7.8**	0.69±0.07	28
**8.0**	1.28±0.07	15
**8.1**	2.40±0.12	8
**8.2**	4.10±0.62	5

### On-column cleavage

**Figure 4:**
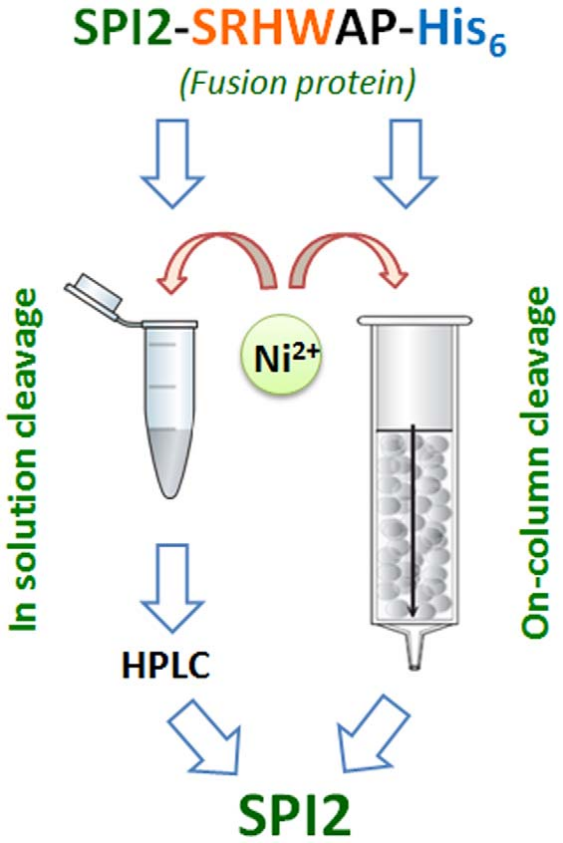
Alternative approaches to Ni(II)-dependent affinity tag cleavage in protein purification. The tag can be removed in solution (left) or when immobilized at the affinity column (right).

**Figure 5:**
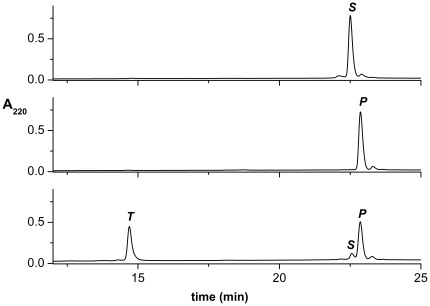
The examples of chromatograms of reaction mixture containing initially 80 µM SPI2-SRHWAP-His_6_ fusion protein loaded on the Ni-NTA-agarose column, incubated with 4 mM NiCl_2_ in 100 mM Hepes buffer, pH 8.2 at 50°C. From top to bottom: control fusion protein (incubated without Ni(II) ions), incubation buffer, and two pooled wash fractions (250 mM imidazole) after 22 h of incubation.

We used the in-solution strategy of the affinity tag cleavage in order to obtain kinetic data for the reaction characterized previously only for model peptides. In terms of practical application, however, it was more important to develop a one-step protein purification method. Therefore, subsequently, we tested the tag removal reaction with the SPI2 fusion protein immobilized on the Ni-NTA column (in brief, the *on-column* cleavage, Fig. 4). The purification of the fusion protein was done as described above, to determine its amount. The purified SPI2-SRHWAP-His_6_ was reloaded on the Ni-NTA agarose column. The effect of temperature on the reaction kinetics was investigated first. 80 µM SPI2-SRHWAP-His_6_ protein immobilized on the column was incubated with 4 mM Ni(II) in 100 mM Hepes buffer, 100 mM NaCl at pH 8.2. The range of temperatures between 28 and 50°C was used. The temperature dependence of the 1^st^ order rate constants is shown in Table 3. Fig. 5 presents chromatograms of control fusion protein (incubated without Ni(II) ions), incubation buffer and two pooled wash fractions (250 mM imidazole) after 22 h of incubation at 50°C. Molecular masses of the collected peaks confirmed the absence of unspecific cleavage. A single peak was observed in the chromatogram of the incubation buffer. This fraction collected after 22 h of incubation contained substantial amounts of the pure SPI2 protein. The HPLC analysis of both wash buffers contained small amounts of SPI2 that were adsorbed at the NTA-agarose column. The total yield of SPI2 purification was nearly 100%. The activity of the native protein obtained in our procedure was only slightly lower from that of the fusion protein, with IC_50_ = 126±28 nM and 76±16 nM, respectively. Therefore the prolonged incubation at an elevated temperature did not have a significant effect on the protein activity. This difference might be also caused by a C-terminal modification of the protein, as was reported previously [22].

**Table 3:** The temperature dependence of the 1^st^ order rate constant and reaction half-time for the on-column SPI2-SRHWAP-His_6_ hydrolysis, at pH 8.2, determined by HPLC.

T [°C]	k ± S.D. (s^−1^×10^−5^)	t_1/2_ (h)
28	0.96±0.37	20
37	1.00±0.63	19
40	2.00±0.09	10
45	4.00±3.0	5
50	6.00±2.0	3

**Figure 6:**
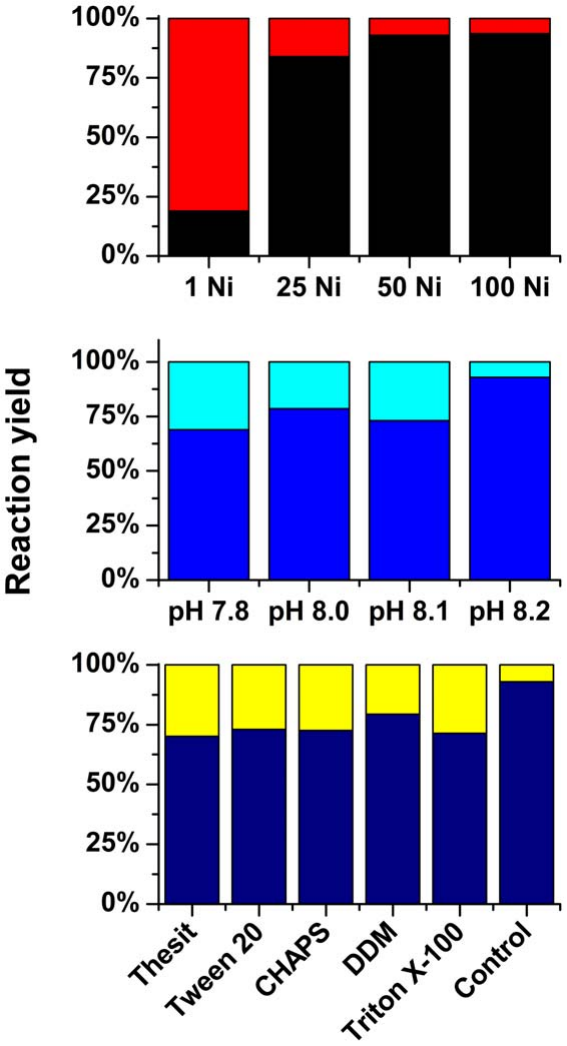
The effect of Ni(II) ions concentration, pH and various detergents on the hydrolysis of SPI2-SRHWAP-His_6_ fusion protein.

The effect of various Ni^2+^ concentrations, from 1 to 100 molar equivalents of the protein, was analyzed next. The fusion protein at concentration of 80 µM immobilized on the column was incubated in 100 mM Hepes at pH 8.2. At one molar equivalent of Ni(II) ions the efficiency of the cleavage did not exceed 20% even after 24 h of incubation at 50°C (Fig. 6). The tag removal efficiency increased with the increasing Ni(II) concentration. There was a difference between the effect of 25-fold and 50-fold molar excess of Ni(II) ions, while the difference between the 50-fold and 100-fold molar excess was marginal. We also studied the dependence of this process on pH in the range of 7.8–8.2 (Fig. 6). The fusion protein (80 µM) was incubated with the 50–fold molar excess of Ni(II) ions in 100 mM Hepes buffer. There were only slight differences in the efficiency of the reaction in the studied pH range, contrary to peptide studies, where the reaction was much more vigorous at higher pH [17]. At pH values above 8.2 Ni(II) ions in a large excess over the target protein increasingly tend to precipitate as nickel hydroxide. Therefore the positive effect of increasing the pH may be canceled by the loss of Ni(II) from the reaction mixture.

Numerous proteins require sophisticated conditions of handling. Among them membrane proteins are especially troublesome, due to their hydrophobic nature. The reaction mechanism presented by us previously suggested that the presence of detergents would not affect the hydrolysis [17]. We therefore incubated the SPI2-SRHWAP-His_6_ fusion protein (80 µM) with 4 mM Ni(II) ions with detergents used commonly for membrane protein solubilization: Thesit, Tween 20, Triton X-100, DDM, and CHAPS [23]. After 24 h incubation at 50°C the incubation buffer and the wash fraction were analyzed by HPLC. The total efficiency of the cleavage reached nearly 80% (Fig. 6).

There is often a need to maintain reductive conditions during the purification process. This particularly concerns those proteins which possess sensitive thiols. Some other proteins require denaturing conditions. The presence of denaturants or reductants affects proteolytic enzymes and inhibits their hydrolytic activity. Therefore we tested our reaction in the presence of GuHCl and separately with 1 mM DTT. The former reagent slightly inhibited the cleavage; however the yield still exceeded 80%. The presence of DTT at a concentration of 1 mM had no effect on the Ni(II)-dependent affinity tag cleavage (data not shown).

### Ubiquitin hydrolysis

**Figure 7:**
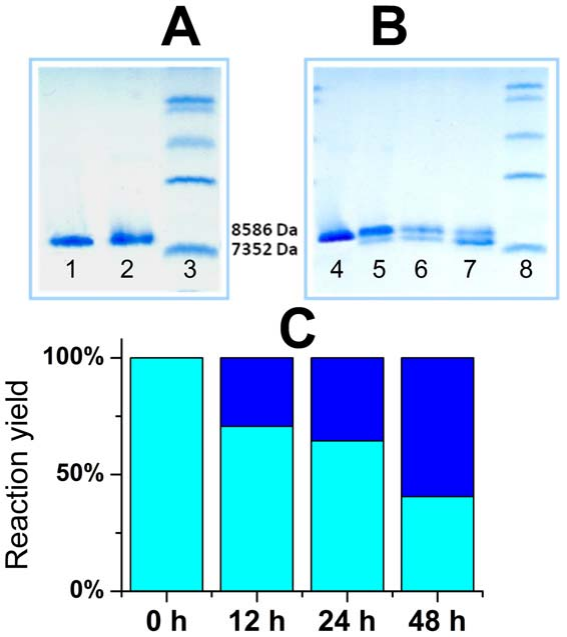
Electrophoretic analysis of Ni(II) hydrolysis of Ubi. (A) native conditions, 100 µM Ubi incubated at 50°C in 20 mM Tris, pH 8.2 for 0 h in the absence of Ni(II) (lane 1), 100 µM Ubi incubated at 50°C for 24 h in the presence of 5 mM Ni(II) (lane 2), low-range protein molecular weight marker (lane 3); (B) denaturating conditions, 100 µM Ubi incubated at 50°C and pH 8.2 in the presence of 5 mM Ni(II) and 5 M GuHCl for 0 h (lane 4), 12 h (lane 5), 24 h (lane 6), and 48 h (lane 7); low-range protein molecular weight marker (lane 8) (C) densitometric analysis of gel (B).

Ubiquitin (Ubi), a highly-conserved eukaryotic protein has wide biotechnological uses [24]. Human Ubi contains a TLHL sequence in positions 66–69, making it a potential target for Ni(II) ions. This sequence is part of the C-terminal β-strand. In order to test the hydrolytic activity of Ni(II) ions toward Ubi we incubated this protein in the presence of Ni^2+^ ions according to several protocols. First, the samples of 100 µM Ubi were incubated in 20 mM Tris buffer with a 5 molar excess of Ni(II) ions, at pH 8.2 and 37°C or 60°C. No cleavage was seen, despite prolonging the incubation up to 5 days. Ubiquitin at a concentration of 100 µM was also incubated in 20 mM Hepes buffer, pH 8.2 with a 25 molar excess of Ni(II) ions. The reaction was controlled by SDS-PAGE. Again, no cleavage was seen after 15 h of incubation at 50°C (Fig. 7). The SPI2-SRHWAP-His_6_ fusion protein was cleaved with good yields under similar conditions. Thereby, it can be assumed that Ubi is resistant to cleavage by Ni(II) ions under non-denaturing conditions applicable to the practical method of affinity tag removal.

In contrast, the presence of 5 M GuHCl made Ubi susceptible to hydrolysis, as demonstrated for 100 µM Ubi incubated with 5 mM Ni(II) at pH 8.2 and 50°C. The SDS-PAGE analysis of samples collected after 12 h, 24 h and 48 h demonstrates clearly the hydrolysis progress (Fig. 7). As predicted, the cleavage occurred specifically at the S65-T66 bond, preceding the Ni(II)-susceptible TLHL sequence. The cleavage site was confirmed by the molecular mass of 7376 Da of the hydrolysis product, compared to 8593 Da of Ubi.

### Confirmation of reaction mechanism using hydroxylamine

**Figure 8:**
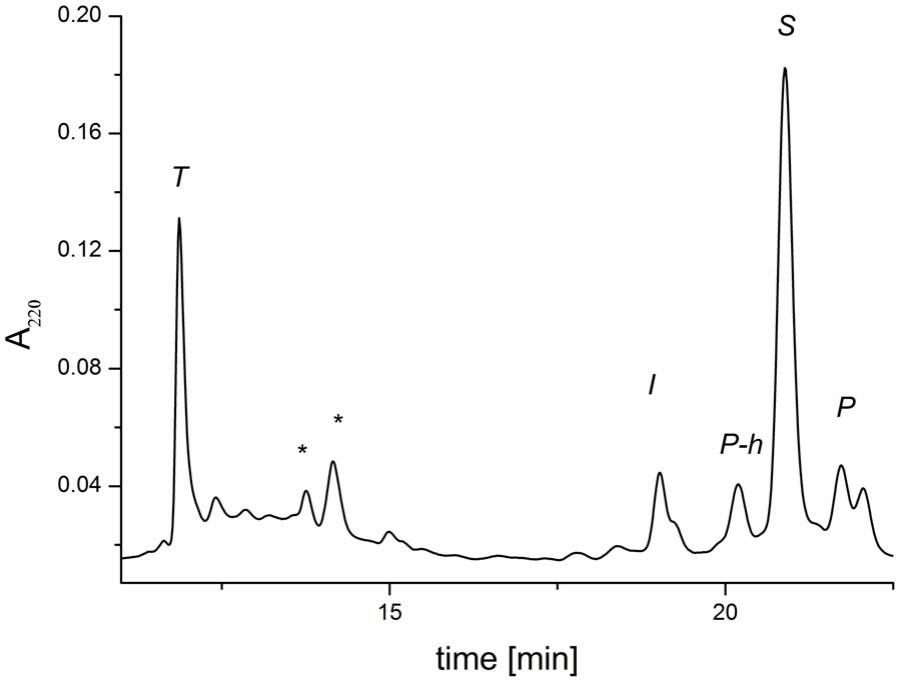
The HPLC chromatograms of the SPI2-SRHWAP-His_6_ fusion protein incubated with Ni(II) ions in the presence of 0.25 M hydroxylamine. Peak labels denote reaction substrate and products, identified using ESI-MS: ***S***, substrate (5868 Da); ***P***, pure SPI2 protein (4310 Da); ***P-h***, SPI2 protein hydroxamate (4326 Da); ***T***, SRHWAP-His_6_ tag (1574 Da); ***I***, the intermediate product of hydrolysis (5868 Da). * impurity peaks which did not exhibit coherent molecular masses.

The requirement of a hydroxyl side chain (Ser or Thr) in the active peptide sequence suggested the involvement of the intermediate ester formation. This nature of the intermediate reaction product was confirmed in peptide studies [17]. In order to make sure that the protein reaction follows the same molecular mechanism, we tested the identity of the intermediate product also for SPI2-SRHWAP-His_6_. The fusion protein was incubated with 5 mM Ni(II) at 37°C. After 6 h of incubation hydroxylamine was added to the final concentration of 0.25 M. This test was based on the susceptibility of ester bonds to cleavage by treatment with hydroxylamine, which does not cleave peptide bonds normally [25]. The formation of a C-terminal hydroxamate of SPI2 following the addition of hydroxylamine to the intermediate product was detected by HPLC/ESI MS analysis (Fig. 8, the product SPI2 mass increased by 16 Da (4325 vs. 4309) upon the hydroxylamine treatment, as expected of a hydroxamate derivative).

### Prevalence of the hydrolytic motif

In order to estimate the danger of encountering a native hydrolytic motif, such as the one found by us previously in C2H2 zinc fingers or human Histone H2A [26], [27], we performed a standard search of the Uniprot database, looking for the prevalence of our hydrolytic motif among 20254 human proteins present in that database [28]. We found 3470 proteins (17%). We can expect that these motifs will be largely present in secondary structure elements, thus being not accessible to nickel hydrolysis. Therefore, the danger of additional cleavage of the recombinant protein is not large.

## Discussion

**Figure 9:**
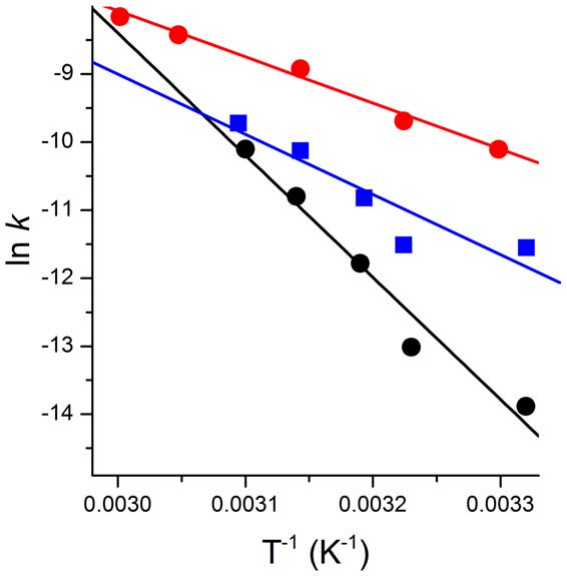
Arrhenius plots, demonstrating the temperature dependence of hydrolysis reaction rates at pH 8.2 for SPI2-SRHWAP-His_6_ fusion protein hydrolysed in-solution (black), and on-column (blue), compared to that of the Ac-GASRHWKFL-amide peptide (red) [**17**] **.** Lines represent linear fits to experimental points.

The results of solution studies of the affinity tag removal from SPI2 can be compared to those obtained previously for peptide models [16], [17]. Qualitatively, we can state beyond doubt that the protein reaction has the same molecular mechanism as the peptide reaction. This is confirmed by the same sequence specificity of cleavage, the same course of reaction, including the ester intermediate (Fig. 8), and the same profile of pH dependence of the rate constant (Fig. 3) as observed previously for the Ac-GASRHWKFL-NH_2_ peptide which provided the active sequence SRHW to the fusion protein. The major difference was that the peptide reacted severalfold faster than the protein under comparable conditions (the lower concentration of the fusion protein was compensated by the excess of Ni(II), so that the relative concentrations of the active species were similar). The Arrhenius plot in Fig. 9 shows that this superiority of the peptide diminished with the increase of temperature. This indicates that the slower protein reaction was due to a less favourable conformation of the active complex within the tag, compared to that of the free peptide. The faster reaction was seen for the on-column hydrolysis, as seen in reaction half-times in Tables 2 and 3, and in Fig. 9. The kinetic parameters of this process cannot be compared directly with those obtained from solution experiments. However, experiments performed in the range of pH, temperatures and nickel to protein ratios demonstrated that the on-column removal of affinity tag proceeds fast enough to be used in protein purification. This option is also operationally superior to the solution reaction, because it simplifies the procedure significantly. Further experiments were therefore performed for this option only.

Membrane channels, receptors and transporters constitute extremely attractive targets for research and industry as potential drug interaction sites. However, the expression and purification of recombinant membrane proteins is very difficult. One problem is constituted by the continuous requirement for detergents during the purification process. These detergents have to be removed before the affinity tag removal since their presence inhibits proteolytic enzymes. We demonstrated that the presence of detergents affects the tag removal efficiency only slightly (Fig. 6). A similar result was obtained for two other typical biochemical reagents, denaturant GuHCl and reductant DTT.

The applicability of our method is based on the assumption that Ni(II) ions can interact with –SRHW-like sequences only at solvent exposed tags. Otherwise we would be posed with unspecific protein cleavage by Ni(II) ions. In a concurrent study we identified one such motif in C2H2 zinc fingers [26]. We verified the crucial assumption that Ni(II) ions would not be able to penetrate protein interiors or distort secondary structure elements using human ubiquitin. This protein naturally possesses the potentially active Thr-Leu-His-Leu sequence. However, despite prolonged incubations in the presence of Ni(II) ions at elevated temperatures no cleavage was observed for the natively folded protein, while Ubi denaturated by GuHCl was hydrolysed (Fig. 7).

As we demonstrated above, our method works best at the elevated temperature and alkaline pH. Therefore, it can be recommended for thermostable proteins, which it can provide with a good efficiency and high purity, in the presence of a range of typical protein buffer components. It can also be used for more sensitive proteins, at a lower temperature or pH, at the cost of prolonged incubation times.

### Conclusions

The SPI2-SRHWAP-His_6_ fusion protein was expressed in *Pichia pastoris* system. Two affinity tag cleavage strategies were tested. The *in-solution* strategy included standard affinity purification, followed by cleavage of the tag in the presence of Ni(II) ions and separation of the cleavage products by HPLC. The *on-column* strategy included cleavage of the tag directly on the Ni-NTA column. For the first time we present the highly specific and efficient chemical method for affinity tag removal in procedures of recombinant protein purification, which can be applied in a wide range of conditions, and in the presence of common biological buffer components.

## Materials and Methods

### SPI2-SRHWAP-His_6_ fusion protein expression and purification

The biologically active recombinant SPI-2 protein, extended C-terminally with the His-tag was previously expressed in a *Pichia pastoris* system [20]. The alternative SPI2-SRHWAP-His_6_ fusion protein was designed in order to verify the title method. The appropriate gene construct was successfully cloned under the control of AOX promoter in a pPICZαB vector (Invitrogen), using standard methods. The fusion protein secreted to the medium was pre-purified by affinity chromatography on Ni-NTA-agarose (Qiagen) in the presence of 20 mM phosphate buffer, pH 7.4, containing 0.5 M NaCl. In a standard procedure, 2 mL of Ni-NTA-agarose was used to purify a 100 mL portion of medium. The fusion protein was then eluted from the column with 250 mM imidazole, pH 7.4, and dialyzed against water overnight in order to remove the excess of salts. Next, the protein was purified by HPLC on a Vydac C18 semipreparative column. The eluting solvent A was 0.1% TFA/water and solvent B was 0.1% TFA/90% acetonitrile/water. A linear gradient from 10% to 40% in 30 min at a flow rate of 2 mL/min was applied, with detection at 220 nm and 280 nm. After elution, the fusion protein was frozen and lyophilized.

### In-solution affinity tag cleavage

The tag removal reaction was conducted at several temperatures (50, 45, 40, 37 and 28°C) and pH values (8.2, 8.1, 8.0, 7.8, 7.5). All of the samples were prepared and then incubated in low adsorption 1.5 ml tubes (Eppendorf LowBind), using a thermoblock (TB-941U, JW Electronic). Typically, 20 µM SPI2-SRHWAP-His_6_ fusion protein was incubated with 0.5 mM Ni^2+^ in 100 mM Hepes buffer. The reaction was stopped by acidification (2% TFA). The samples were then refrigerated, typically for up to several hours, before injection to the HPLC system (Breeze, Waters) on the C18 column (ACE, 250×4.6 mm). The linear gradient used was 1% per min of 10–25% buffer B, followed by 0.1% per min of 25–26% buffer B. The flow rate was 1 mL/min. The molecular masses of collected HPLC peaks were measured using ESI MS (Q-Tof1, Micromass).

### On-column affinity tag cleavage

The SPI2-SRHWAP-His_6_ fusion protein was loaded on Ni-NTA-agarose (Invitrogen), according to the manufacturer's instruction. For studying the temperature effect, 80 µM protein was incubated in 100 mM Hepes buffer, pH 8.2 with 4 mM Ni^2+^ at 28, 37, 40, 45 and 50°C without shaking. Samples were incubated in 1.5 mL low binding Eppendorf vials in a thermoblock. After the incubation the buffer was collected. Then the remaining substrate and the cleaved off SRHWAP-His_6_ domain were washed from the column with 250 mM imidazole, pH 7.4. The samples were separated using HPLC (Breeze, Waters) on the C18 column (ACE, 250×4.6 mm). The linear gradient from 10% to 25% solvent B was used. The flow rate was 1 mL/min. The molecular masses of collected HPLC peaks were measured using ESI MS (Q-Tof1, Micromass). For the examination of the effect of pH (range 7.8–8.2) and Ni(II) ions concentration (0.8–8.0 mM metal salt, corresponding to 1–100 molar equivalents of protein), 100 µL of the resin with the immobilized fusion protein, was mixed with 200 µL of 100 mM Hepes buffer. The final fusion protein concentration was 80 µM. These experiments were performed at 50°C. The effects of 1 mM dithiotreitol (DTT), 0.6 M guanidine chloride (GuHCl) and 1% detergents: Triton X-100, Tween 20, Thesit, n-dodecyl-β-D-maltoside (DDM), CHAPS were studied with the same fusion protein concentration in 100 mM Hepes buffer, pH 8.2 containing 4 mM Ni(II).

### Test of activity of SPI2

The activity of the SPI2 protein after affinity tag cleavage (24 h incubation at 50°C) and SPI2-SRHWAP-His_6_ (without incubation) were measured against Proteinase K (PK), using casein substrate. The reactions were performed in triplicate using 5.1 µM succinylated casein, 250 ng of Proteinase K and 33–333 nM of either inhibitor. The reactions also contained 0.006% picrylsulfonic acid solution (TNBS) and 50 mM sodium borate, pH 8.0. All reactions were carried out in 96 well microtitre plates. They were initiated by the addition of enzyme and incubated at 37°C with shaking for 30 min. The absorption at 405 nm (A_405_), proportional to the concentration of the reaction product [21] was determined in a multi-detection microplate reader (Synergy HT, Biotek). The blank reaction contained all components except casein. Net A_405_ was calculated by subtracting the blank from the A_405 nm_ of the reaction. The IC50 values were calculated using ORIGIN 8.5 software.

### C-terminal modification of the SPI2 target protein during the Ni(II)-dependent affinity tag cleavage

The fusion protein (100 µM) was incubated in 1 M Hepes buffer pH 8.2 with 5 mM Ni(II) at 37°C for 6 hours. Then hydroxylamine was added to a final concentration of 0.25 M. The pH of the sample was adjusted to 6.0. The reaction mixture was subsequently incubated for 48 hours at 37°C without shaking, and then analyzed by HPLC under conditions described above. All the peaks were collected and identified by ESI MS (Q-Tof1, Micromass)

### Ni(II)-dependent cleavage of human ubiquitin

Two samples of 100 µM human ubiquitin, Ubi K48R, (Sigma) were incubated in 20 mM Tris buffer, pH 8.2, with 0.5 mM Ni(II), separately at 37°C and 60°C. Samples were incubated for up to 5 days. The reaction was controlled by SDS-PAGE in a Tris-glycine buffer using a Coomassie stained 4–20% double layer gel. A separate 100 µM Ubi sample was also incubated at 50°C in a 20 mM Hepes buffer, pH 8.2 containing 2.5 mM Ni(II). Analogous experiments were performed under denaturating conditions. 100 µM human Ubi was incubated with 5 mM Ni(II) and 5 M GuHCl at 50°C. Samples collected after 12, 24 and 48 h were analyzed by SDS-PAGE. The control samples contained Ubi incubated in 20 mM Tris buffer pH 8.2 without Ni(II) ions and with or without GuHCl.
