# Correction: Application of Ni(II)-Assisted Peptide Bond Hydrolysis to Non-Enzymatic Affinity Tag Removal

**DOI:** 10.1371/annotation/69b27043-7cf8-4980-a078-57d7704f2b8b

**Published:** 2012-08-03

**Authors:** Edyta Kopera, Agnieszka Belczyk-Ciesielska, Wojciech Bal

There were errors in the funding section. The correct funding information is as follows: This study was financed in part by Polish National Center for Research and Development, grant no. KB/138/13749/IT1-B/U/08 and in part by the project "Metal-dependent peptide hydrolysis. Tools and mechanisms for biotechnology, toxicology and supramolecular chemistry” carried out as part of the Foundation for Polish Science TEAM program, co-financed from European Regional Development Fund resources within the framework of Operational Program Innovative Economy. The funders had no role in study design, data collection and analysis, decision to publish, or preparation of the manuscript.

